# Detection of Influenza and Other Respiratory Pathogens by RT-qPCR and Characterization by Genomic Sequencing Using ILI/SARI Hospital-Based Sentinel Surveillance System

**DOI:** 10.3390/v17081131

**Published:** 2025-08-18

**Authors:** Charity A. Nassuna, Fahim Yiga, Joweria Nakaseegu, Esther Amwine, Bridget Nakamoga, Noel Ayuro, Nicholas Owor, David Odongo, Jocelyn Kiconco, Thomas Nsibambi, Samuel Wasike, Ben Andagalu, Chelsea Harrington, Adam W. Crawley, Julius Ssempiira, Ray Ransom, Amy L. Boore, Barnabas Bakamutumaho, John T. Kayiwa, Julius J. Lutwama

**Affiliations:** 1 Uganda Virus Research Institute, National Influenza Center, Department of Arbovirology, Entebbe, Uganda; cnassuna@uvri.go.ug (C.A.N.); fyiga@uvri.go.ug (F.Y.); jbwanika@uvri.go.ug (J.N.); estheramwine1122@gmail.com (E.A.); brid8t33@gmail.com (B.N.); nayuro@uvri.go.ug (N.A.); nowor@uvri.go.ug (N.O.); dodongojr@uvri.go.ug (D.O.); jkiconco@uvri.go.ug (J.K.); bbakamutumaho@uvri.go (B.B.); jlutwama@uvri.go.ug (J.J.L.); 2 US Centers for Disease Control and Prevention, Division of Global Health Security, Atlanta, GA 30333, USA; meq8@cdc.gov (T.N.); hnp0@cdc.gov (S.W.); twr9@cdc.gov (B.A.); lur1@cdc.gov (C.H.); qyi4@cdc.gov (A.W.C.); snz7@cdc.gov (J.S.); rlr1@cdc.gov (R.R.); gdn6@cdc.gov (A.L.B.)

**Keywords:** influenza, SARS-CoV-2, RSV, other respiratory viruses, metagenomic next-generation sequencing, Uganda

## Abstract

Limited surveillance and laboratory testing for non-influenza viruses remains a challenge in Uganda. The World Health Organization (WHO) designated National Influenza Center (NIC) tested samples from patients with influenza-like illness (ILI) and severe acute respiratory infections (SARIs) during August 2022–February 2023. We leveraged the influenza sentinel surveillance system to detect other respiratory viruses (ORVs). Samples were tested using the US Centers for Disease Control and Prevention (CDC) influenza and SARS-CoV-2 multiplex and the FTD^TM^ Respiratory Pathogens 21 assays using real-time reverse transcription polymerase chain reaction (RT-qPCR). A total of 687 (ILI = 471 (68.6%) and SARI = 216 (31.4%) samples were tested. The median age was 2 years (IQR: 1–25) for ILI and 6 years (IQR: 1–18) for SARI case definitions (*p*-value = 0.045). One or more respiratory pathogens were detected in 38.7% (*n* = 266) of all samples; 33 (12.4%) were selected for metagenomics sequencing and 8 (3%) for SARS-CoV-2 targeted sequencing. Respiratory pathogens were detected by sequencing in 23 of 33 (69.7%) samples. Our study provides insight into the usefulness of this surveillance system in conducting virological testing for other viruses and provides tools and evidence to monitor patterns and characteristics of viruses causing ILI/SARI, which will guide public health decisions and interventions in Uganda.

## 1. Introduction

Respiratory viruses are responsible for significant global morbidity and mortality, affecting the younger and older populations disproportionately [1,2,3]. Acute lower respiratory infections are the leading causes of morbidity and mortality in children under 5 years globally, with developing countries contributing 97% of that burden [4]. The most common causes of respiratory infections are human respiratory syncytial virus (HRSV), human rhinovirus (HRV), human adenovirus (HAdV), influenza, human metapneumovirus (hMPV), enteroviruses (EVs), and parainfluenza types [5]. The relationships between clinical symptoms and respiratory infections have been described frequently, but virus detection provides more specific information on the correlations between clinical symptoms and specific infections. For bacteria and fungi, it is difficult to determine their relevance to the symptoms. Nevertheless, such assays provide epidemiological data that can be useful for treatment planning and prevention of infections.

In 2007, the US Centers for Diseases Control and Prevention (CDC) started a collaboration with the Uganda Virus Research Institute (UVRI), under the Ministry of Health (MoH), to establish a hospital-based sentinel surveillance network for influenza across Uganda to support early detection, determine the epidemiology, seasonality, and burden of circulating and novel influenza viruses. Past surveillance data in Uganda have reported influenza positivity rates of 10–13% [6], but the prevalence of other respiratory viruses (ORVs) is probably under-reported [7]. This may be attributed to their exclusion from the routine testing algorithm and the limitations of multiplex PCR panels to a few respiratory pathogens. Other studies prior to COVID-19 in Uganda (May 2019 to March 2020) show incidence rates of respiratory symptoms of 2.2 times higher in children than in adults (568.4 vs. 254.2 cases per 1000 person-months, relative risk (RR) 95% CI (1.8, 2.8)) [8]. The same study showed respiratory disease incidence rates in adults were comparable to previously reported rates for similarly aged adults (20–54 years) globally.

With the emergence of the COVID-19 pandemic, the need for surveillance of ORVs has been highlighted, and the WHO recommended leveraging Global Influenza Surveillance and Response Systems (GISRS) for surveillance of SARS-CoV-2, RSV, and other common respiratory viruses [9].

Our overall objective was to highlight the importance of leveraging and strengthening the capacity of National Influenza Centers (NICs) for the preparedness and response to not only epidemic influenza, SARS-CoV-2, and RSV but also ORVs by RT-qPCR detection and characterization by genomic sequencing. Also, we sought to assess the feasibility of conducting surveillance for multiple respiratory viruses to identify and understand their epidemiology among ILI and SARI patients with acute respiratory infections.

## 2. Materials and Methods

### 2.1. Study Site, Specimen, and Data Collection

The hospital-based influenza surveillance network is composed of 16 sentinel sites, which are geographically spread out around Uganda [9]. Patients were enrolled using two case definitions; for outpatients, the ILI case definition for an acute respiratory infection was a measured fever of ≥38 °C and a cough with onset within the last 10 days. SARI case definition for an acute respiratory infection was a subjective or measured temperature of ≥38 °C and a cough with onset in the last 10 days requiring hospitalization [10]. Each weekday during the surveillance period, nasal pharyngeal and/or oral pharyngeal (NP/OP) swabs were collected during August 2022 and February 2023, by nurses and clinical officers, from the first patients of all age groups with ILI (maximum 10 patients), and from all patients with SARI, as previously reported [6]. The FLOQSwabs^®^ Flocked Swabs—COPAN were used to collect the samples and placed in Universal Transport Medium^TM^ (UTM^®^)—COPAN and stored frozen in liquid nitrogen tanks (Taylor Wharton 35 VHC, USA) until their shipment to the WHO-designated NIC laboratory at UVRI, where they were kept at −80 °C until testing.

### 2.2. RNA Extraction and Multiplex RT-qPCR

Nucleic acid extraction and RT-qPCR testing using CDC’s Influenza and SARS-CoV-2 Multiplex Assay were performed following the CDC protocol as previously described [9]. All samples were also tested for ORVs using the FTD^TM^ Respiratory Pathogens 21 assay (Siemens Healthineers, Erlangen, Germany), which is a qualitative in vitro nucleic acid amplification test (NAAT) for the detection and differentiation of specific viral and bacterial nucleic acids using multiplex rRT-PCR, following the manufacturer’s recommendations. The tested respiratory pathogens using the FTD^TM^ Respiratory Pathogens 21 assay included human rhinovirus (HRV), human coronaviruses (HCoVs) NL63, 229E, OC43, and HKU1, human parainfluenza viruses (HPIVs) 1 through 4, human metapneumoviruses (HMPVs) A and B, human bocavirus (HBoV), human respiratory syncytial viruses (HRSVs) A and B, human adenovirus (HAdV), enterovirus (EV), and human parechovirus (HPeV). An internal control was also included. Positive HRSV samples were further tested for RSV subtypes A and B using the CDC Respiratory Syncytial Virus Multiplex Real-Time RT-PCR following the manufacturer’s recommendations. Briefly, 5 μL of RNA were added to AgPath-ID^TM^ One-Step RT-PCR master mix (ThermoFisher Scientific, Waltham, MA, USA), [5.0 μL of nuclease-free water, 0.5 μL RSV forward primer, 0.5 μL RSV reverse primer, 0.5 μL FAM probe, 12.5 μL 2× PCR mastermix, and 1.0 μL enzyme mix] for a total reaction volume of 25.0 μL. RT-qPCR amplification conditions were as follows: reverse transcription at 45 °C for 10 min; PCR initiation denaturation at 95 °C for 15 s; PCR amplification (45 cycles) at 95 °C for 15 s and 55 °C for 60 s. All fluorescence data (FAM) were collected during the 55 °C incubation step.

### 2.3. Sequencing and Analysis

All SARS-CoV-2 RT-qPCR positives with a Ct value < 30 were selected for sequencing using the SWIFT Normalase^®^ Amplicon Panel-SARS-CoV-2 protocol and loaded on the Illumina iSeq 100 using iSeq 100 i1 v2 reagents (Illumina, San Diego, CA, USA) [2 × 150 cycles]. Sequence reads were aligned against the SARS-CoV-2 Wuhan-Hu-1 reference (GenBank accession NC_045512.2) using bwa-mem2 v2.2.1, and the consensus genome was called using sam2consensus (min depth = 10). SARS-CoV-2 variants were called using Nextclade v2.14.0. ORV PCR positive samples with Ct values < 30 were selected for sequencing on the Illumina MiSeq v3 reagent kit (Illumina, USA) [2 × 300 cycles] following a shotgun metagenomics approach and using the NEBNext^®^ Ultra^TM^ II Directional RNA Library Prep Kit for Illumina^®^ (New England Biolabs, Inc, Ipswich, MA, USA). Quality trimming (Q30) was performed using trim galore v0.6.10 [11], and taxonomic screening was performed using Kraken v2.1.3 [12] and a standard database (https://benlangmead.github.io/aws-indexes/k2 accessed on 9 April 2024). Taxonomic binning results (for known respiratory pathogens with more than five reads) informed the selection of reference genomes for alignment, which was performed using bwa-mem2 v2.2.1 [13]. Consensus genomes were called using sam2consensus (min depth of 5), and their taxonomies were then determined using blastn v2.14.1 [14] against the RefSeq database.

### 2.4. Data Management and Statistical Analysis

Data management was performed in EpiInfo 7.1.0.6, and analysis was conducted using R statistical software version 4.2.3. Descriptive analysis was conducted for categorical and numerical data and presented as frequencies, proportions, means, and medians. Pearson’s chi-square and Wilcoxon rank sum tests were used to assess differences in frequencies between groups. Statistical significance was set at *p*-value ≤ 0.05.

This activity was reviewed by CDC, deemed not research, and was conducted consistently with applicable federal law and CDC policy.

## 3. Results

### 3.1. Demographic Characteristics of Patients

The study population comprised 687 patients with 471 (68.6%) ILI and 216 (31.4%) SARI cases. The median age of the enrolled patients was 2 years (IQR: 1–25) for ILI and 6 years (IQR: 1–18) for SARI (Table 1). The ILI category had more females, 318 (68%), than males, 152 (32%), whereas the SARI category had more males, 114 (53%), than females, 101 (47%); 61 (13%) ILI patients had one or more demographic characteristics missing.

### 3.2. Pathogen Detection and Identification by RT-qPCR

**Figure 1:**
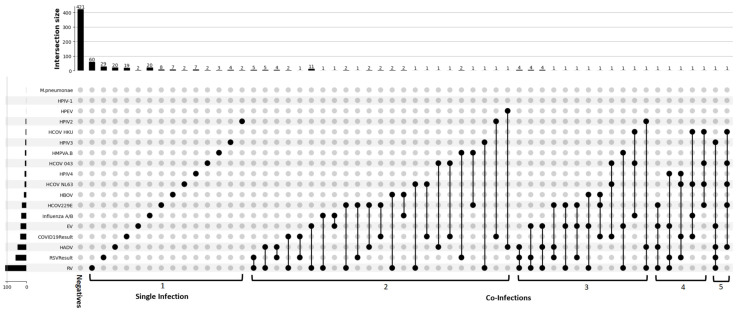
Detection of single and mixed pathogens. (The round black dots denote the type of pathogen(s) detected on the Y-axis against single or co-infections on the horizontal axis.) The scale on the top shows the number of single or co-infection cases detected along the specific column.

**Figure 2:**
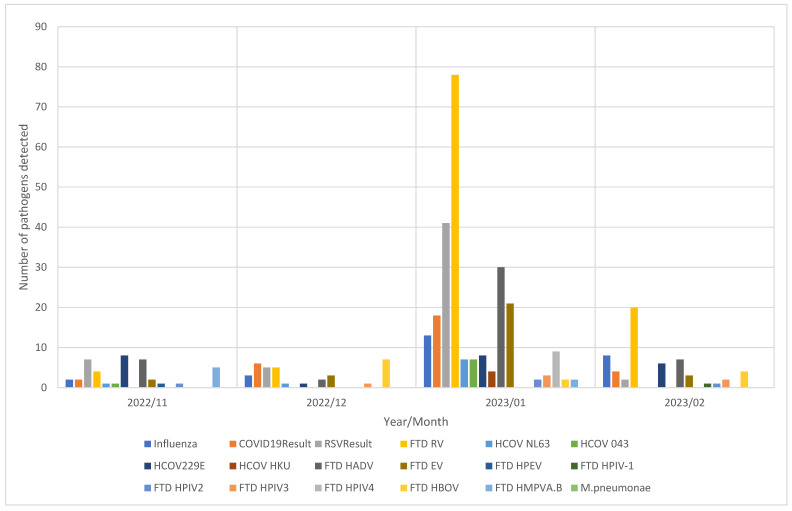
Number of positive respiratory pathogens detected by year and month.

Of the 687 samples tested, 266 (38.7%) tested positive for one or more respiratory pathogens. A single pathogen was detected in 186 (69.9%) specimens, and two or more pathogens were detected in 80 (30.1%) of all positive samples. The four most detected single pathogens in order of highest to lowest were HRV (*n* = 60; 8.7%), followed by HRSV (*n* = 29; 4.2%), HADV (*n* = 20; 2.9%), influenza A/B (*n* = 20; 2.9%), and the least detected single pathogens were EV (*n* = 2; 0.3%), HCoV 043 (*n* = 2; 0.3%), HCoV NL63 (*n* = 2; 0.3%), and HPIV2 (*n* = 2; 0.3%) (Figure 1). Out of 471 ILI cases, 165 (35.0%) were positive, of which 120 (72.7%) were confirmed to have a single infection. Of the 216 SARI cases, 100 (46.3%) were positive, and 66 (66%) were confirmed to have a single infection. 55 RSV-A and RSV-B infections were detected, with RSV-A (33/55; 60.0%) being detected more than RSV-B (22/55; 40%). The majority of the pathogens were detected in January 2023, with HRV being the most detected (Figure 2).

### 3.3. Prevalence of Multiple Infections

Co-infections were detected in 80 (11.6%) samples, with HRV being the most frequent virus co-detected with other viruses, and the most frequent combinations being HRV + EV (*n* = 11; 1.6%), followed by HRV + HAdV (*n* = 5; 0.7%) and HRV + HRSV (*n* = 5; 0.7%) (Supplementary Table S1). The frequency of mixed infections decreased with the increase in the number of combinations.

### 3.4. Genomic Sequencing

Out of 19 (2.8%) SARS-CoV-2 PCR positive specimens, 8 samples (SARI = 6 and ILI = 2), with Ct values < 30 were selected for genomic sequencing, and all were Omicron variants with sub-lineages of BQ.1 (*n* = 1), BQ.1.1 (*n* = 1), XBB.1 (*n* = 3), XBB.3.2 (*n* = 1), BA.2 (*n* = 1), and XBB.1.5 (*n* = 1). The sequences and metadata of the genomes were uploaded into the GISAID repository with accession numbers ranging from EPI_ISL_17293039 to EPI_ISL_17293045 and EPI_ISL_18075887.

Out of 266 (38.7%) PCR-positive samples, a total of 33 (12.4%) samples (Ct value < 30) were selected for shotgun metagenomics sequencing, and respiratory pathogens were detected in 23 (69.7%) samples. A single respiratory pathogen was identified in 19 of 23 (82.6%) samples, while co-infections were found in 4 of 23 (17.4%) samples. A comparison of virus detection between RT-qPCR and genomic sequencing identified the same respiratory pathogens in 15 of 23 (65.2%), whereas 8 of 23 (34.8%) were discordant, notably a SARS-CoV-2 being reported as HCoV 229E by RT-qPCR (GISAID accession EPI_ISL_19333808). Metagenomic sequencing also identified 6 other commensal and opportunistic respiratory pathogens not tested for by the FTD^®^ respiratory pathogen 21 kit, and these included *H. influenzae*, Human betaherpes viruses 5 and 7, *Moraxella catarrhalis*, *Streptococcus* sp., and *Neisseria* sp.

Out of 17 HRV PCR positives (Ct value < 30) selected for sequencing, only 9 (52.9%) were detected by metagenomics sequencing. Furthermore, partial viral genomes could only be reliably assembled (minimum depth of 5) in 6 out of 9 cases. This lower detection rate could have been due to sequencing depth limitations coupled with low viremia, which could have compromised the sensitivity compared to RT-qPCR. The 6 HRVs were characterized as HRV-A (*n* = 3), HRV-B (*n* = 2), HRV-C (*n* = 1), and one being a co-infection of HRV-A and HRV-C. The partial genomes were uploaded to the GenBank sequence repository under accession numbers PQ226459, PQ226460, PQ226461, PQ226462, PQ226463, and PQ226464. Other pathogens detected by metagenomic sequencing and for which partial genomes were reliably reconstructed included 1 *Haemophilus influenzae* (GenBank accession number PQ435196), 1 Human mastadenovirus B (GenBank accession number PQ226466), and 2 HMPV identified as strain A (GenBank accession numbers PQ226458 and PQ226465). A near-complete genome (99.9%) of Human coronavirus NL63 (GenBank accession no. PQ226467) was reconstructed, and phylogenetic and mutation analysis did not show any significant differences with other previously circulating NL63 strains elsewhere in the world.

## 4. Discussion

This study demonstrated the effectiveness of using the existing NIC surveillance program under the WHO GISRS network to expand the detection of ORVs, other than influenza, SARS-CoV-2, and RSV, using rapid and sensitive multiplex real-time RT-qPCR assays. This enables the determination of the epidemiology and detection of respiratory infections, which are major causes of symptoms in ILI and SARI cases. By using RT-qPCR, our study detected respiratory viruses in 38.7% of all enrolled cases, with a prevalence level close to that previously reported in Uganda [7]. Other studies, however, have reported higher prevalence levels of over 50% in comparison to our study [15]. The reason could be due to differences in sample size and duration of the studies. Children less than 6 years of age accounted for most of the ILI cases in comparison to children less than 10 years old for the SARI cases, as previously described [6].

Our study detected similar trends of influenza cases during the study period compared to other studies performed in Uganda [16]. In contrast to a previous study [8], our virus detection rate of 29.8% for co-infections was twice as high. The reason for the difference could be due to more pathogens (*n* = 21) being tested in our study compared to the previous study (*n* = 12). However, similar prevalence levels were observed in this study for the three most detected single respiratory viruses, which were HRV (8.7%), followed by HRSV (4.2%) and lastly HAdV (2.9%), whereas the least detected single viruses were EV, HCoV 043, HCoV NL63, and HPIV2 (0.3%), compared to a similar study [7].

Prior to COVID-19 in Uganda, respiratory disease incidence rates were 568.4 cases per 1000 person-months in children and 254.2 cases per 1000 person-months in adults; however, after COVID-19 lockdown measures were implemented, these rates declined to 256.0 and 160.8 cases per 1000 person-months, respectively [8]. The COVID-19 national lockdown in Uganda included closure of schools, businesses, international airports, roads between districts, as well as increased masking and handwashing. These non-pharmaceutical public health interventions targeting COVID-19 reduced influenza incidence and severity [8].

Of the three routinely tested viruses, HRSV was the most detected, followed by SARS-CoV-2 and lastly influenza during the study period. The high proportion of HRSV in our study is similar to that reported in children from other countries [17,18,19,20,21,22]. Both types of RSV A and B co-circulated, with RSV A being the most dominant. Influenza AH1pdm09 was predominant over influenza AH3 of influenza A types. Influenza B/Victoria was the only influenza B virus genotype detected.

Rhinovirus (HRV) was the most detected respiratory virus that was reported [23,24,25], whereas adenovirus (HAdV) was the third most detected virus in our study. Consistent with prior literature, adenovirus and rhinovirus are etiologies of respiratory infection in asymptomatic cases, with specifically adenovirus infections being common in young children [26,27]. Adenovirus co-infections were the most common infections in our study, as previously reported [7], and it has been suggested that the asymptomatic adenovirus infection in young children might not be the cause of the disease [28]. HRSV, that is, the main etiologic agent of lower respiratory tract infections in children, has been reported to have a prevalence of 14% to 34% and is particularly very common in infants less than one year [19,29,30]. In contrast, the prevalence of HRSV was lower in our study, probably due to the age differences in study participants, with the overall mean age in our study being 13 years and the median age being 4 years. However, HRSV was the second most detected single virus after rhinovirus in our study, with 4.2% prevalence. Only three (HPIV2, HPIV3, and HPIV4) of the four HPIV types were detected in our study, with the most detected being HPIV4, compared with a previous study [7].

While RT-qPCR allowed for fast and targeted detection of specific pathogens with high sensitivity, high-throughput sequencing provided a broader view of the pathogen landscape. Our genomic investigations identified and characterized SARS-CoV-2 as the Omicron variant with various sublineages. Additionally, we identified other viral and non-viral respiratory pathogens such as *H. influenzae*, Human betaherpes viruses 5 and 7, *Moraxella catarrhalis*, *Streptococcus* sp., and *Neisseria* sp., emphasizing the importance of metagenomics in detecting uncommon pathogens. Metagenomic sequencing is such a powerful diagnostic technology and requires careful analysis and interpretation, as the presence of a genus or species is not indicative of pathogenesis.

We failed to identify a pathogen in some PCR-positive samples, probably because metagenomic sensitivity is limited by biological and technical factors. This lower detection rate could have been limited by pipeline parameters and read coverage [31], due to sequencing depth limitations, probably because some of these agents are present through transient carriage, which could have compromised their sensitivity compared to RT-PCR. Also, a possible explanation for the discrepancies in the results obtained by the two methods is that, by sequencing, the reads obtained are aligned to all reference sequences in the gene bank databases. In contrast, qPCR detection is limited by using primers, which are designed to target sections of the gene. In addition, qPCR is more vulnerable to the presence of inhibitors in the samples; therefore, the used conditions might not be optimal for all PCR primers, resulting in lower abundances.

While qPCR represents a fast, cost-effective, and sensitive approach to detect pathogens, the technique is dependent upon known target sequences that contain conserved primer target sites. Such preconditions are not always satisfied, and shotgun metagenomic sequencing is sometimes required for broader and more accurate evaluation. The discrepancies can be confirmed using Sanger sequencing, but this was not performed in the present study and represents one of the study’s limitations.

The results of this multi-pathogen detection study strongly suggest that influenza sentinel surveillance capacity under the WHO GISRS network can be leveraged and strengthened for the surveillance of other respiratory pathogens. Using ILI and SARI case definitions among all age groups, the system was able to detect one or more respiratory pathogens in more than 30% of the specimens tested using RT-qPCR.

This work, however, had some limitations. The study period was limited to four months during a period when COVID-19 activity was low; therefore, additional years of surveillance data will contribute to a better understanding of the epidemiology of ORVs. Although a substantial proportion of ILI and SARI are associated with bacterial pathogens, only one respiratory bacterium, *M. pneumoniae*, was tested in our study by the FTD^TM^ Respiratory Pathogens 21 assay, and all samples were negative. Nevertheless, our study aimed to leverage the existing influenza surveillance system to detect ORVs during the period of low COVID-19 activity.

From a public health perspective, the Uganda Ministry of Health is strengthening and implementing the electronic Integrated Disease Surveillance and Response (eIDSR) system by integrating ILI/SARI and Acute Febrile Illness (AFI) sentinel surveillance into the National Integrated Surveillance Systems (NISS) framework; therefore, our results can be used to guide the understanding of the respiratory virome disease surveillance, where there are still limitations in surveillance and laboratory testing capacity for non-influenza viruses. The NISS framework will aim to improve epidemic intelligence and early warning alert and response (EWAR) for diseases and conditions of epidemic and public health importance.

## 5. Conclusions

We demonstrated the usefulness of the WHO GISRS influenza sentinel surveillance platform in the collection of comprehensive information on the etiologies of SARI and ILI cases during the COVID-19 pandemic period in Uganda. This study also provides insights into the virome of acute-respiratory diseases and the usefulness of genomic characterization of circulating pathogens from individuals with ILI and SARI as evidence to monitor prevalence, patterns, and characteristics of viruses causing acute respiratory illness, which will be used to guide public health decisions and interventions.

The study also highlights the importance of using both PCR and NGS methods for gene detection and identification. While both methods identify some genes with similar abundance, they also complement each other by identifying genes that are missed by other methods. This combination of both methods can provide more accurate and comprehensive results and, careful analysis and optimization of the threshold setting can help improve the reliability of the results. Overall, considering the strengths and limitations of each method and using them in a complementary way is essential for optimal results.

## Figures and Tables

**Table 1:** Sex and age characteristics among ILI and SARI patients.

Characteristic	Overall, N = 687	ILI, N = 471	SARI, N = 216	*p*-Value
	*n* (%)	*n* (%)	*n* (%)	
Sex				**<0.001 ^1^**
Female	419 (61%)	318 (68%)	101 (47%)	
Male	266 (39%)	152 (32%)	114 (53%)	
Unknown	2	1	1	
Age (Years)				**0.045 ^2^**
N	625	410	215	
Median (IQR)	4 (1, 24)	2 (1, 25)	6 (1, 18)	
Range	0, 85	0, 62	0, 85	
Missing	62	61	1	

^1^ Pearson’s Chi-squared test; ^2^ Wilcoxon rank sum test.

## Data Availability

All sequence data generated and used in the study are available in GISAID and GenBank.

## Supplementary Materials

The following supporting information can be downloaded at https://www.mdpi.com/article/10.3390/v17081131/s1, Table S1: Frequency of detections for co-infections.
