# Supplementary material for: Detection of Influenza and Other Respiratory Pathogens by RT-qPCR and Characterization by Genomic Sequencing Using ILI/SARI Hospital-Based Sentinel Surveillance System
Source: Viruses. 2025 Aug 18;17(8):1131. doi: 10.3390/v17081131 (PMC12390689; doi:10.3390/v17081131)
Supplement: Supplementary file 1 [file viruses-17-01131-s001.zip › viruses-3710889_Supplimentary.pdf]

SUPPLEMENTARY INFORMATION FOR

Detection of Influenza and Other Respiratory Pathogens by RT-qPCR and Characterization by Genomic Sequencing Using ILI/SARI Hospital-Based Sentinel Surveillance System

Charity A Nassuna [1], Fahim Yiga [1], Joweria Nakaseegu [1], Esther Amwine [1], Bridget Nakamoga [1], Noel Ayuro [1], Nicholas Owor [1], David Odongo [1], Jocelyn Kiconco [1], Thomas Nsibambi [2], Samuel Wasike [2], Ben Andagalu [2], Chelsea Harrington [2], Adam W Crawley [2], Julius Ssempiira[2], Ray Ransom [2], Amy Boore [2], Barnabas Bakamutumaho [1], John T Kayiwa [1]* and Julius J Lutwama [1].

[1]Uganda Virus Research Institute
[2]US Centers for Disease Control and Prevention
* Correspondence: author: jkayiwa@uvri.go.ug; Tel: +256774313662

Supplementary table

Table S1. Frequency of detection for co-infections

Double Infections	***Number Positive***	***Percentage (n=687)***
HRV/EV	11	1.6
HRV/HADV	5	0.7
HCoV 043/HADV	1	0.2
HRSV/HRV	5	0.7
HRSV/HADV	4	0.6
Flu/HBOV	2	0.3
COVID-19/HCoV 229E	2	0.3
COVID-19/RV	2	0.3
HRSV/HMPV	2	0.3
HRV/HBOV	2	0.3
HRV/HCoV 229E	2	0.3
HADV/HCoV 229E	2	0.3
Flu/EV	1	0.2
Flu/HRV	1	0.2
COVID-19/HCoV 043	1	0.2
COVID-19/HCoV NL63	1	0.2
COVID-19/HPIV2	1	0.2
COVID-19/HRSV	1	0.2
HADV/HPEV	1	0.2
HCoV 229E/HMPV	1	0.2
HRSV/HCoV 229E	1	0.2
HRV/HCoV NL63	1	0.2
HRV/HPIV3	1	0.2
Total	**51**	
Triple Infections	**Number Positive**	
HRV/HADV/EV	4	0.6
HRSV/HRV/EV	4	0.6
HRSV/HRV/HADV	4	0.6
HRSV/HADV/HCoV 299E	1	0.2
HRSV/HCoV 229E/EV	1	0.2
Flu/HCoV 043/HCoV HKU	1	0.2
COVID-19/HCoV 299E/HBOV	1	0.2
COVID-19/HCoV NL63/HCoV 043	1	0.2
HRSV/HCoV 229E/HADV	1	0.2
HRV/EV/HBOV	1	0.2
HRV/EV/HMPVA.B	1	0.2
HRV/HADV/HPIV2	1	0.2
HRV/HCoV 229E/EV	1	0.2
Total	**22**	
Quadruple Infections	**Number Positive**	

Flu/COVID-19/HCoV NL63/HCoV HKU	1	0.2
COVID-19/HRSV/HCoV NL63/HPIV4	1	0.2
HCoV NL63/HCoV 043/HCoV 229E/HCoV HKU	1	0.2
HRSV/HRV/EV/HPIV4	1	0.2
HRV/HCoV 229E/HADV/EV	1	0.2
Total	**5**	
Five Infections	**Number Positive**	
HCoV NL63/HCoV 043/HCoV229E/HCoV HKU/HADV	1	0.2
HRSV/HRV/HADV/EV/HPIV3	1	0.2
Total	**2**	
Overall total	**80**	
